# Biomarker analysis in stage III–IV (M0) gastric cancer patients who received curative surgery followed by adjuvant 5-fluorouracil and cisplatin chemotherapy: epidermal growth factor receptor (*EGFR*) associated with favourable survival

**DOI:** 10.1038/sj.bjc.6604936

**Published:** 2009-03-03

**Authors:** J-S Kim, M-A Kim, T M Kim, S-H Lee, D-W Kim, S-A Im, T-Y Kim, W H Kim, H-K Yang, D S Heo, Y-J Bang, K-U Lee, K-J Choe, N K Kim

**Affiliations:** 1 Department of Internal Medicine, Seoul National University Hospital, 28 Yongon-dong, Chongno-gu, Seoul, 110-744, Republic of Korea; 2 Department of Pathology, Seoul National University Hospital, 28 Yongon-dong, Chongno-gu, Seoul, 110-744, Republic of Korea; 3 Department of Surgery, Seoul National University Hospital, 28 Yongon-dong, Chongno-gu, Seoul, 110-744, Republic of Korea; 4 Cancer Research Institute, Seoul National University College of Medicine, Seoul, 110-744, Republic of Korea

**Keywords:** gastric cancer, adjuvant chemotherapy, pharmacogenetics, epidermal growth factor receptor, thymidine phosphorylase, excision repair cross-complementing gene 1

## Abstract

The aim of this study was to analyse the impact of epidermal growth factor receptor (*EGFR*), thymidylate synthase (*TS*), dihydropyrimidine dehydrogenase (*DPD*), thymidine phosphorylase (*TP*), aurora kinase (ARK) A/B, and excision repair cross-complementing gene 1 (*ERCC1*) on the efficacy of adjuvant chemotherapy with 5-fluorouracil and cisplatin (FP) after curative gastric resection. Normal and cancer tissue were separately obtained from gastrectomy samples of 153 patients with AJCC stage III–IV (M0) who subsequently treated with adjuvant FP chemotherapy. *TS*, *DPD*, *TP*, *ERCC1*, and ARK proteins were measured by immunohistochemistry (IHC). *EGFR* expression was investigated using a standardized IHC with the *EGFR* PharmDx assay. Amplification of *EGFR* gene was analysed using fluorescent *in situ* hybridisation (FISH). In multivariate analysis, stage, ratio of positive to removed lymph nodes, and *EGFR* expression were significant prognostic factors for overall survival. Patients with higher *EGFR* expression had better overall survival than those with lower expression (relative risk: 0.475 (95% confidence interval, 0.282–0.791, *P*=0.005). Low *EGFR* expression might be a predictive marker for relapse in curative resected stage III–IV (M0) gastric cancer patients who received adjuvant FP chemotherapy.

The prognostic and predictive roles of epidermal growth factor receptor (*EGFR*) expression in gastric cancer remain controversial, and the reported frequencies of *EGFR* expression are varied in gastric cancer (Gamboa-Dominguez *et al*, 2004; Matsubara *et al*, 2008b). In the past, high levels of *EGFR* were reported as a poor prognostic factor for overall survival (OS) in resectable gastric cancer patients who did not receive chemotherapy (Garcia *et al*, 2003; Galizia *et al*, 2007). In contrast, high levels of *EGFR* were reported as a positive prognostic factor in patient group who received 5-fluorouracil (FU)-containing chemotherapy (Al-Batran *et al*, 2008a; Matsubara *et al*, 2008a). Thymidylate synthase (*TS*), thymidine phosphorylase (*TP*), and dihydropyrimidine dehydrogenase (*DPD*) are known key enzymes in the metabolism of 5-FU and play a role in resistance to fluoropyrimidines. Thymidylate synthase expression level is presumed to influence response to 5-FU-containing chemotherapy, although *TS* is not unanimously recognised as a determinant of 5-FU sensitivity (Danenberg, 2004; Park and Lenz, 2006). Thymidine phosphorylase catalyses the reversible phosphorylation of thymidine to thymine 2-deoxyribose-1-phosphate, and increases the conversion of 5-FU to its active metabolites, which play an important role in the inhibition of *TS* (Tahara *et al*, 2004). Dihydropyrimidine dehydrogenase is the initial and rate-limiting enzyme in the catabolism of 5-FU. Although the role of *DPD* levels in tumours have not been firmly established as a prognostic factor for clinical responsiveness, there is ample evidence that a *DPD* deficiency is associated with severe toxicity after 5-FU administration (van Kuilenburg, 2004). Expression of the excision repair cross-complementing gene 1 (*ERCC1*) may play a role in human tumours because it is essential for nucleotide excision repair and influences genomic instability (Chen *et al*, 2000). For example, low gene expression levels of *ERCC1* were associated with a superior response to 5-FU and cisplatin chemotherapy (FP) in primary gastric cancer (Metzger *et al*, 1998), and *ERCC1* protein expression levels were found to be inversely associated with response. Excision repair cross-complementing gene 1 may possibly have a role in the clinical resistance to platinum compounds in gastric cancer patients (Kwon *et al*, 2007). The Aurora kinases, a family of mitotic regulators, have received much attention as potential targets of new drugs (Warner *et al*, 2006) and in their association with chemoresistance to platinum agents (Yang *et al*, 2006). However, none of these markers have previously been evaluated in an adjuvant setting in high-risk gastric cancer patients undergoing potentially curative surgery.

## Materials and methods

### Study population

From the database of Seoul National University Hospital, we identified a total of 5387 patients who underwent gastrectomy between November 1995 and November 2003. Patients with a diagnosis of histologically proven gastric cancer, who received a curative gastrectomy with D2 dissection and adjuvant chemotherapy consisting of 5-FU and cisplatin were identified. Cisplatin (60 mg m^−2^ as 15 min i.v. infusion) followed by 5-FU (1200 mg m^−2^ as 12 h continuous i.v. infusion for 4 days) was given in 21-day cycles. The following eligibility criteria were used for patients’ enrollment: age <75 years; free from distant metastatic disease; stages III_A_, III_B_, and IV (only non-metastatic cases, T4 N1-3 and T1-3 N3; *AJCC Cancer Staging*, 6th edition); no prior chemotherapy or radiotherapy; World Health Organisation (WHO) performance status ⩽2; adequate baseline organ function, defined as WBC count ⩾4000 cells per ml, platelet count ⩾100 000 cells per ml, serum bilirubin level ⩽1.5 mg ml^−1^, serum creatinine level ⩽2.0 mg/100 ml, serum albumin level ⩾3.0 mg/100 ml, no severe uncontrolled comorbidities (e.g., myocardial infarction in the last 12 months); no second malignancies; and informed consent.

### Patient follow-up

In the absence of symptoms, physical examination was performed every 3–4 months for 5 consecutive years. Follow-up assessment consisted of physical examination, a complete blood count, liver function test, chest radiography, and abdominal ultrasound or CT scan, every 3–6 months for 5 years. Toxicities were graded according to NCI-CTC version 2. The site and date of the first relapse and the date of death, if the patient died, were recorded. Survival was calculated from beginning of surgery until the last follow-up or death from any cause; patients who were alive at the last follow-up were censored at that time. Patients who were taken off study or who died before progression were censored at the time when they were taken off from the study. Survival data were confirmed either by medical records or by the death reports from the Korea Central Cancer Registry.

### Tissue sampling

Cancerous and adjacent normal tissues were obtained from the surgically resected and paraffin-embedded primary gastric cancer specimens of the patients. Samples were examined histologically for the presence of tumour cells.

### Tissue microarray methods

Core tissue biopsy specimens (2 mm in diameter) were obtained from individual paraffin-embedded gastric tumours (donor blocks) and arranged in a new recipient paraffin block (tissue array block) using a trephine apparatus (Superbiochips Laboratories, Seoul, Korea). Each tissue array block contained up to 60 cases, allowing a total of 153 pairs to be contained in six array blocks. An adequate case was defined as one with a tumour occupying more than 10% of the core area. Each block contained an internal control consisting of non-neoplastic gastric mucosa from adjacent tissue. Sections of 4 *μ*m were cut from each tissue array block, deparaffinised, and dehydrated. As shown earlier, staining results obtained from different intratumoural areas in various cancers correspond with each other (Lee *et al*, 2007), and a core was sampled in each case.

### Immunohistochemistry (IHC)

**Figure 1:**
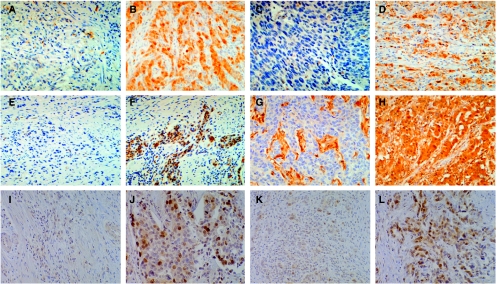
Typical examples of positive immunohistochemical staining. **A**: *TS* (−) **B**: *TS* (2+) **C**: *DPD* (−) **D**: *DPD* (2+). **E**: *ERCC1* (−) **F**: *ERCC1* (2+) **G**: *TP* (−) **H**: *TP* (2+). **I**: *ARK1* (−) **J**: *ARK1* (2+) **K**: *ARK2* (−) **L**: *ARK2* (2+). In the immunohistochemical (*IHC*) analysis of thymidylate synthase (*TS*), dihydropyrimidine dehydrogenase (*DPD*), excision repair cross-complementing gene 1 (*ERCC1*) and thymidine phosphorylase (*TP*), the degree of IHC reactivity was graded from 0 to 3+ according to the cytoplasmic staining.

Immunohistochemical staining for *TS*, *TP*, and *ERCC1* was performed using an ABC method (labelled biotin) after a microwave antigen retrieval process. Mouse monoclonal antibodies of *TS*, *TP*, and *ERCC1* were obtained from Neomarkers, Fremont, CA, USA. Staining intensity and stained tumour cell percentages were measured. Stained cell percentage was multiplied by the staining intensity (0–3+), which resulted in an IHC score ranging from 0 to 300 for each cell type, as described previously (Ren *et al*, 2004). Rabbit polyclonal antibodies to *ARK-1* and *ARK-2* were obtained from Santa Cruz Biotechnology Inc., CA, USA. Immunohistochemical staining for *EGFR* was performed on the tissue microarray slides using the *EGFR* PharmDx kit (DAKO Cytomation) according to the manufacturer's instructions. The positive samples were classified further into 1+, 2+, and 3+, based on their staining intensity. The highest staining intensity of all tissue cores from the same tumour was scored as the final immunohistochemical result. Pathologists unaware of clinical outcomes independently scored the immunohistochemical staining. We determined the appropriate cutoff value for the IHC score of *TS*, *TP*, and *ERCC1* with the highest sensitivity and specificity by the receiver operating characteristic (ROC) curve method. Typical staining intensities for *TS*, *DPD*, *ERCC1*, *TP*, ARK-1, and ARK-2 are shown in Figure 1.

### *EGFR* fluorescent *in situ* hybridization (FISH) analysis

*EGFR* (7p12) gene amplification was determined using a DNA probe set (LSI EGFR/CEP 7; Vysis, Downers Grove, IL, USA) consisting of a SpectrumOrange-labelled *EGFR* (locus)-specific probe and a SpectrumGreen-labelled probe that hybridises to the centromeric region of chromosome 7 according to protocols described elsewhere (Mitsui *et al*, 2007).

### Statistical analysis

Statistical analysis was performed on a personal computer using SPSS 12.0K for windows (SPSS Inc. Chicago, IL, USA). The survival rate was calculated using the Kaplan–Meier method, and a statistical analysis was performed using log-rank test. Multivariable analysis of prognostic factors was conducted by Cox proportional hazards model; *P*<0.05 was considered statistically significant.

## Results

Between 1 November 1995 and 30 November 2003, a total of 153 patients were eligible for the study. The demographic characteristics are outlined in Table 1. The group consisted of 108 men (70.6%) and 48 women (29.4%) with a median age of 52.0 (range: 15–72) years. Median follow-up duration was 72.9 months (range: 2.0–135.0 months). Demographic and clinical data are described in our earlier report in detail.

### Expression of *EGFR*, *TS*, *TP*, *DPD*, and *ERCC1* proteins

Epidermal growth factor receptor expression using the PharmDx kit revealed a score of 0 in 29 patients (19.3%), 1+ in 56 patients (37.3%), 2+ in 56 patients (37.3%), and 3+ in 9 patients (6.0%). Epidermal growth factor receptor expression had no significant association with clinicopathologic variables, such as age, gender, stage, PS, Lauren's classification, and Borrmann type and differentiation. Fluorescent *in situ* hybridization analysis of *EGFR* was evaluable in a total of 135 patients, which showed high polysomy in three patients (2.2%) and amplification in four patients (3.0%). Epidermal growth factor receptor expression had no significant correlation with FISH positivity. The expression levels of *TS*, *TP*, and *ERCC1*, as determined by IHC, in cancer tissue were not significantly different in relapsed and non-relapsed patients. These expression levels were not different between stages. However, expression of *TP* and *ERCC1* was significantly higher in cancerous tissue than in normal tissue (*P*<0.0001). No significant correlation between *TP* and *ERCC1* levels was found in cancerous and normal tissues. Dihydropyrimidine dehydrogenase expression levels in cancerous tissues were not significantly different in relapsed and non-relapsed cases; however, *DPD* levels were significantly lower in cancerous tissue (*P*<0.0001). We could not analyse the correlation between cancerous and normal tissue *DPD* levels, because normal tissues expressed *DPD* with uniform intensity and distribution; *ERCC1* expression was positively correlated with *EGFR* expression (*P*=0.047, ANOVA test). Expression of *ARK1* and *ARK2* were positive in 68.1 and 69.3%, respectively.

### Prognostic factors

**Figure 2:**
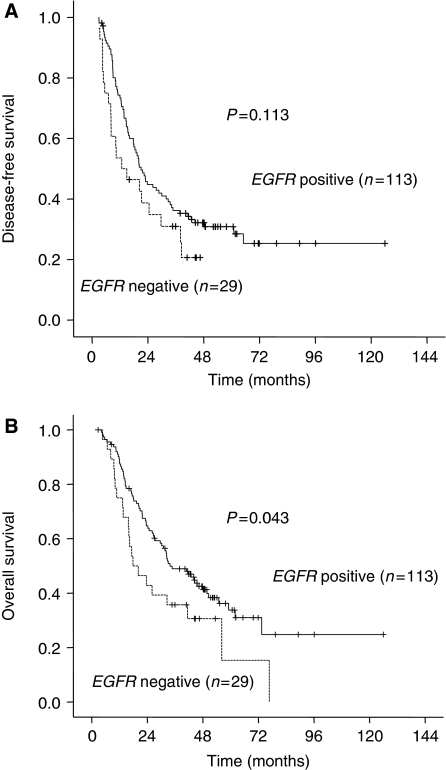
Kaplan–Meier estimates of (**A**) disease-free survival and (**B**) overall survival of the patients according to the expression of epidermal growth factor receptor (*EGFR*).

**Figure 3:**
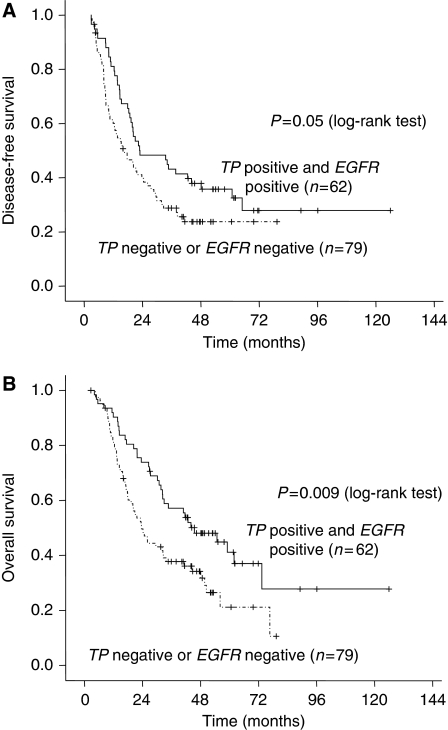
Kaplan–Meier estimates of (**A**) disease-free survival and (**B**) overall survival of the patients according to the expression of thymidine phosphorylase (*TP*) and epidermal growth factor receptor (*EGFR*).

**Figure 4:**
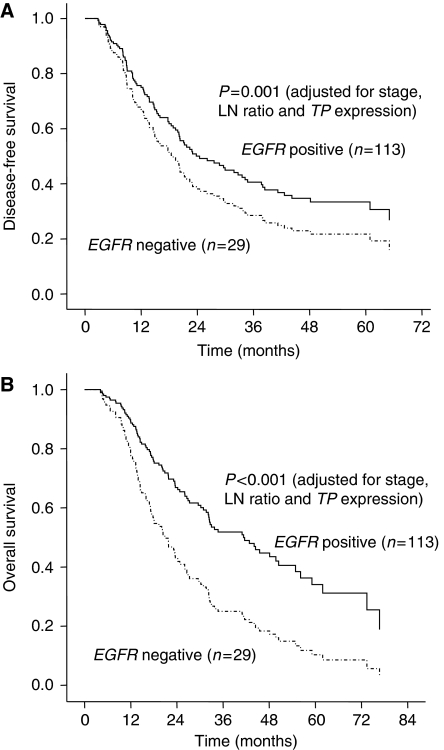
Kaplan–Meier estimates of (**A**) disease-free survival and (**B**) overall survival of the patients according to the expression of epidermal growth factor receptor (*EGFR*) after being adjusted for the stage, LN ratio and thymidine phosphorylase (*TP*) expression.

We performed univariate analyses of biomarkers associated with relapse and survival (Table 2). Epidermal growth factor receptor and *TP* expressions were significant prognostic factor for OS in univariate analysis (Table 2, Figure 2). However, expression of *TS* and *ARK1* were not significantly associated with relapse and survival. Parameters with *P*-values of ⩽0.10 were included in the multivariate analysis using the Cox proportional hazards. The positive LN ratio was an independent prognostic marker for both disease-free survival (DFS) and OS. Relapse and survival were significantly influenced by stage, the ratio of invaded, resected lymph nodes, and by the expression of *TP*, *ERCC1*, and *EGFR* (Table 3). Low *EGFR* expression was an independent biomarker for predicting poor OS in multivariate analysis. The relationship between the coupled expression of *TP* and *EGFR* and clinical outcomes are shown in Figure 3A and B. Patients with higher *TP* and *EGFR* expression showed longer DFS (26.0 *vs* 16.0 months, *P*=0.05) and OS (45.3 *vs* 23.6 months, *P*=0.009) than the other patients. After adjustments for stage, LN ratio, and *TP* expression, *EGFR* expression was remained as a significant prognostic factor for both DFS and OS (Figure 4A and B). The DFS and OS were not significantly influenced according to the grade of *EGFR* expression or *EGFR* amplification or polysomy detected by FISH.

## Discussion

Epidermal growth factor receptor expression was a prognostic factor in our study, but the prognostic role of *EGFR* in gastric cancer needs to be further elucidated. Some reports showed that high levels of *EGFR* expression are associated with more distant metastasis, more advanced stage, and poorer OS (Garcia *et al*, 2003; Gamboa-Dominguez *et al*, 2004; Galizia *et al*, 2007). In advanced gastric cancer patients treated mainly with 5-FU or cisplatin-based chemotherapy, lower expression of *EGFR* mRNA than the cutoff value was a strong predictor of poor survival by multivariate analysis (Matsubara *et al*, 2008a). Interestingly, the authors also showed that high *DPD* and *ERCC1* expressions were significant predictors of poor survival. Another recent report showed that *EGFR* expression was a positive prognostic factor in patients with AGC (Al-Batran *et al*, 2008a), which was a retrospective analysis from the phase III trial comparing 5-FU, folinic acid plus either oxaliplatin *vs* cisplatin (Al-Batran *et al*, 2008b). There has also been evidence suggesting that cytotoxic chemotherapy is more effective among patients with high *EGFR* expression than in those with low *EGFR* expression (Ceppi *et al*, 2006; Vallbohmer *et al*, 2006). The prognostic and predictive roles of *EGFR* expression in gastric cancer thus remain controversial. Differences in *EGFR* expression among these studies may also be attributed to the lack of an established immunohistochemical scoring system commonly used to evaluate gastric cancer. Other prognostic variables, such as insulin-like growth factor type 1 receptor (Matsubara *et al*, 2008c) and class I histone deacetylase expression (Weichert *et al*, 2008) might also have confounding interactions.

In gastric cancer patients, higher *TP* expression was reported in cancerous tissues compared with the adjacent normal tissues (Maeda *et al*, 1996; Tanigawa *et al*, 1996; Kakeji *et al*, 1999) by IHC and ELISA. These studies indicated that *TP* expression is closely correlated with cancer invasion, haematogenous metastasis, lymph node metastasis, venous invasion, lymphatic invasion, and microvascular invasion. In our study, IHC scores from cancerous tissue were significantly higher than those of normal tissue. Higher *TP* expression might be a prognostic marker for OS, but the role of *TP* expression in gastric cancer also needs to be further elucidated.

Many reports have indicated that *DPD* activities are unaltered in gastric cancer (Ishikawa *et al*, 1999; Terashima *et al*, 2002). However, it was also reported that gastric carcinomas have significantly higher *DPD* activities than normal mucosa (Nakata *et al*, 2004). Another report showed that *DPD* expression in cancer cells, but not in stromal cells, could predict the efficacy of 5-FU chemotherapy in patients with T3 gastric carcinoma (Hisamitsu *et al*, 2004). In our study, the IHC scores of cancerous tissues were significantly lower than those of normal tissues, and no significant difference was observed between relapsed and non-relapsed patients in terms of IHC scores.

Decreased *ERCC1* expression was associated with a superior response to 5-FU/cisplatin in primary intact gastric cancer patients (Metzger *et al*, 1998). The degree of *ERCC1* protein expression was found to be inversely associated with this response, which is potentially relevant to clinical resistance to platinum compounds (Kwon *et al*, 2007). In patients with curatively resected gastric cancer, it was shown that increased *ERCC1* expression was correlated with improved outcome (Baek *et al*, 2006). In patients with completely resected non-small-cell lung cancer, *ERCC1*-negative tumours showed greater benefit from cisplatin-based adjuvant chemotherapy compared with *ERCC1*-positive tumours (Olaussen *et al*, 2006). Reports concerning *ERCC1* in resected lung cancer suggest that *ERCC1* overexpression may improve treatment outcome by reducing DNA mutations during cancer progression (Simon *et al*, 2005; Lee *et al*, 2008).

In this study, low *ERCC1* expression was associated with poor survival, but *ERCC1* expression level was also closely associated with *EGFR* expression level. After multivariate analysis, the impact of *ERCC1* on survival disappeared. To our knowledge, the *ERCC1* protein expression between cancer and normal tissues has never been compared, although a report suggested that no significant difference exists in *ERCC1* mRNA expression (Warnecke-Eberz *et al*, 2004). Further prospective studies will be needed to resolve this issue.

Earlier studies evaluated the expression of pharmacogenomic markers as a group. For example, patients with low *TS*, *TP*, and *DPD* gene expression showed prolonged survival over patients expressing high levels of these genes (Salonga *et al*, 2000). The main differences between this and the current study are (1) analysis of mRNA *vs* protein, (2) colorectal cancer *vs* gastric cancer, and (3) a metastatic or disseminated setting *vs* an adjuvant setting. No study has been conducted on the evolution of these pharmacogenomic markers during cancer progression in an adjuvant setting. Therefore, further pharmacogenomic studies on adjuvant treatments are required.

In conclusion, high expression of *EGFR* might be a good predictive marker of relapse and survival in curatively resected stage III–IV (M0) gastric cancer patients who received adjuvant 5-FU and cisplatin chemotherapy. Both *EGFR* expression, the coupled expression of *TP* and *EGFR*, and the LN ratio might be useful predictive markers for patient survival. On the other hand, there was no relationship in this study between clinical outcome and the pharmacogenetic markers reported in earlier studies, such as *TS*, *DPD*, and *ERCC1*. This suggests that these markers might not correlate with chemosensitivity to the FP treatment in gastric cancer patients. Further investigation is necessary, using prospective analysis of a larger cohort in a randomised controlled trial of adjuvant chemotherapy consisting of fluoropyrimidine and platinum agents.

## Figures and Tables

**Table 1:** Characteristics of patients and tumours

	**Patients**	
**Characteristics**	**Number**	**%**
All patients	153	
		
*Age (years)*		
Median	52	
Range	15–72	
		
*Sex*		
Male	105	68.6
Female	48	31.4
		
*ECOG performance status*		
0–1	132	91.5
2	13	8.5
		
*Operation*		
Subtotal gastrectomy	61	39.9
Total gastrectomy	92	60.1
		
*Location*		
Proximal	30	19.6
Distal	123	80.4
		
*Pathology*		
Adenocarcinoma	135	88.2
Signet ring cell carcinoma	18	11.8
		
*Lauren classification*		
Intestinal	44	28.8
Diffuse	86	56.2
Mixed	19	12.4
		
*Borrmann type*		
1	1	0.7
2	13	8.5
3	95	62.1
4	44	28.8
		
*Stage* a		
III_A_	51	33.3
III_B_	32	20.9
IV	70	45.8

Abbreviation: ECOG=Eastern Cooperative Oncology Group.

a *American Joint Committee on Cancer Staging manual*, 6th edition.

**Table 2:** Univariate analyses of clinical prognostic factors (*P*-values)

		**Disease-free survival**		**Overall survival**	
**Factors**	**Number of patients**	**RR of relapse and 95% CI**	***P*-value**	**RR of dying and 95% CI**	***P*-value**
*TS*			0.712		0.153
<25	77	1		1	
⩾25	74	0.927 (0.620–1.387)		0.725 (0.467–1.127)	
					
*TP*			0.10		0.043
<25	66	1		1	
⩾25	85	0.714 (0.477–1.070)		0.638 (0.413–0.986)	
					
*ERCC1*			0.060		0.051
<17.5	65	1		1	
⩾17.5	86	0.677 (0.451–1.017)		0.644 (0.414–1.001)	
					
*EGFR PharmDx*			0.115		0.045
Negative	29	1		1	
1+ to 3+	113	0.676 (0.415–1.101)		0.605 (0.370–0.988)	
					
*ARK1*			0.139		0.297
Negative	45	1		1	
Positive	96	1.385 (0.899–2.134)		0.791 (0.509–1.229)	
					
*ARK2*			0.101		0.067
Negative	46	1		1	
Positive	104	0.712 (0.474–1.069)		0.678 (0.448–1.028)	
					
*EGFR FISH*			0.775		0.440
Negative	111	1		1	
Positive	18	0.918 (0.510–1.652)		0.779 (0.413–1.469)	

Abbreviations: ARK=aurora kinase; CI=confidence interval; EGFR=epidermal growth factor receptor; ERCC1=excision repair cross-complementing gene 1; TP=thymidine phosphorylase; TS=thymidylate synthase; RR=relative risk.

Relative risk adjusted for stage. If the relative risk is >1, the relative risk can be thought as the average increased risk of relapse or dying compared with the reference group. The group with the ratio equal to 1 is the reference group.

**Table 3:** Multivariate analyses of clinical prognostic factors

		**Disease-free survival**		**Overall survival**	
**Factors**	**Number of patients**	**RR of relapse and 95% CI**	***P*-value**	**RR of dying and 95% CI**	***P*-value**
*Positive LN/resected LN*			0.041		0.029
<0.3	45	1		1	
0.3–0.7	75	0.941 (0.538–1.646)		0.858 (0.470–1.566)	
>0.7	33	1.857 (0.925–3.728)		1.780 (0.865–3.663)	
					
*Stage*			0.072		0.016
III_A_	51	1		1	
III_B_	32	1.599 (0.890–2.873)		1.669 (0.885–3.145)	
IV	70	1.874 (1.060–3.314)		2.452 (1.331–4.519)	
					
*EGFR PharmDx*			0.051		0.005
0	29	1		1	
1+ to 3+	113	0.609 (0.370–1.002)		0.475 (0.282–0.791)	
					
*TP expression*					0.077
<25	66			1	
⩾25	85			0.681 (0.445–1.042)	

Abbreviations: RR=relative risk, CI=confidence interval.

A backward likelihood ratio approach was used to select factors for multivariate analysis.

If the RR is >1, the relative risk can be thought as the average increased risk of relapse or dying compared with the reference group. The group with the ratio equal to 1 is the reference group. *P*-value is based on log-rank test.
